# Gait assessment as a functional outcome measure in total knee arthroplasty: a cross-sectional study

**DOI:** 10.1186/s12891-015-0525-2

**Published:** 2015-03-22

**Authors:** Jeeshan Rahman, Quen Tang, Maureen Monda, Jonathan Miles, Ian McCarthy

**Affiliations:** UCL Institute of Orthopaedics and Musculoskeletal Science, London, UK; Royal National Orthopaedic Hospital, London, UK; Biomedical Instrumentation Group, Pedestrian Accessibility and Movement Environment Laboratory, UCL, London, UK

**Keywords:** Total knee arthroplasty, Motion analysis, Knee joint kinematics, Mobility

## Abstract

**Background:**

The aim of the study was to assess gait in total knee arthroplasty (TKA) patients, using a technique that can to be used on a routine basis in a busy orthopaedic clinic.

**Methods:**

A total of 103 subjects were recruited: 29 pre-op TKA patients; 17 TKA patients at 8 weeks post-op; 28 TKA patients at 52 weeks post-op; and 29 age-matched controls. Inertial measurement units (IMUs) were used to assess gait. Limb segment angles, knee angle and temporal parameters of gait were calculated. Specific gait parameters were quantified, and data analysed using MANOVA and discriminant analysis.

**Results:**

The gait of TKA patients as a group was only slightly improved at 12 months when compared with the pre-operative group, and both groups were significantly different to controls in several variables. Knee flexion range in stance was the most important variable in discriminating between patients and controls; knee flexion range in swing was the only variable that showed a significant difference between pre- and post-operative patients. When considered individually, only 1/29 patient was within the normal range for this variable pre-operatively, but 9/28 patients were within the normal range 12 months post-operatively.

**Conclusions:**

Even after 12 months after surgery, many TKA patients have not improved their gait relative to pre-operative patients. Routine gait assessment may be used to guide post-operative rehabilitation, and to develop strategies to improve mobility of these patients.

## Background

Total knee arthroplasty (TKA) is the treatment of choice for end stage knee osteoarthritis. In the year 2011, 79,516 primary TKA procedures were performed in the UK [1]. The number of TKA procedures has tripled since 1990, and it is predicted that a further four-fold increase can be expected in the USA by 2030 [2], and presumably a similar increase can be expected in the UK.

Survival rates for implants are generally good [1,3,4], and are comparable with those of total hip arthroplasty (THA). Despite this, satisfaction with TKA is less than that for THA. 81% of patients were satisfied with their knee surgery outcome [5,6], whereas 91% of THA patients were reported to be satisfied with their surgery [6]. Improvements in SF-12 and comparable Oxford joint specific scores are greater in THA compared with TKA [6]. A number of risk factors have been identified for poor outcome after TKA [5,7,8].

When patients are referred to an orthopaedic clinic with knee pain, a detailed history will be taken, and the knee will be examined for deformity, range of motion, tenderness to palpation and integrity of the ligaments and menisci. In the immediate post-operative period, physiotherapy is instigated, and the patient is discharged when they start mobilising safely and the pain is under control. Post-operatively, patients are seen in out-patient clinics (typically at 8 and 52 weeks post-surgery), at which times the wounds are examined, patients are assessed for pain and passive range of movement, and radiographs taken to assess implant position and alignment. Although knee osteoarthritis has a significant impact on patient mobility, there is no formal assessment of walking ability or mobility during routine clinical assessment, although it may be assessed informally. However, in order to understand the reasons for poor outcome and patient dissatisfaction with total knee arthroplasty, and to optimise mobility post-surgery, it could be very useful to study objective measures of function in TKA patients, so that such measures could be used to guide post-operative rehabilitation.

Gait analysis after total knee arthroplasty has been assessed to two systematic reviews over the past few years [9,10]. These have shown consistently reduced total range of motion in the knee, and reduced range of flexion during stance. There are also indications of altered knee kinetics, with only a third of TKA patients in the studies exhibiting a biphasic pattern of sagittal plane moments. More recently, similar results have been reported for reduced knee angle during stance, but detailed musculoskeletal modelling has shown that the forces and extension moments developed by the quadriceps are reduced in early stance in TKA [11]. Differences in gait characteristics between TKA and THA have been described [12], where TKA patients had significantly lower walking speed than THA patients. It has also been shown that TKA patients walk slower than comparable patients with a unicompartmental implant [13].

Although gait analysis has been used as a research tool in TKA [14-16], there are few studies of objective gait assessment being used as a routine functional assessment in the management of patients with knee osteoarthritis. Traditional laboratory-based gait assessment is time consuming and expensive, requiring the patient to attend a specialist laboratory. External markers are placed on the body, and subjects are then required to walk between a row of cameras and usually over force plate; data are recorded by computer for subsequent post-processing and analysis. The whole process, from preparing the patient for the measurement to final data output can take half a day. This is not feasible for the volume of patients undergoing knee arthroplasty. However, alternative assessments of gait are being developed.

The use of flexible electrogoniometry to assess knee angle in TKA subjects has been reported [17,18]; knee excursion was reported to be reduced in TKA patients up to 18 months after surgery, and the authors proposed that the technique was simple and reproducible, and would be suitable as an outcome measure for research and audit purposes [17]. Spatio-temporal assessments of gait have been shown to be useful in knee OA [19]. We have recently explored the use of inertial measurement units (IMUs) to assess gait in ageing [20] and knee OA [21]. These studies have shown IMUs to be accurate and reproducible in the measurement of joint and limb segment range of motion. Knee flexion range during stance was a useful parameter to discriminate between knee OA patients and age-matched controls with high sensitivity (0.783) and specificity (0.914), and was a much better predictor than knee flexion during swing [21]. IMUs have been used in a comparison of fixed- and mobile-bearing knee replacements [22], and were able provide outcome measures to differentiate the behaviour of these two types of bearing surfaces in older and younger patients.

IMUs are comparatively easy to use, requiring no specialist facilities, and with the potential to be used within a busy clinic or rehabilitation unit. The aim of this study was to evaluate the use of IMUs in busy pre- and post-operative outpatient clinics for patients with TKA. The hypothesis was that pre-operative patients would exhibit abnormal gait patterns, and that post-operatively patients would show an improvement in gait pattern by 12 months.

## Methods

Participants who were undergoing or had recently undergone knee replacement were recruited when they attended out-patient clinics at the Royal National Orthopaedic Hospital. All participants had a radiological diagnosis of knee osteo-arthritis in combination with their clinical history. The degree of osteo-arthritis was not quantified other than functional limitation and pain with regards to decision about surgery. Measurements were performed either pre-operatively or at 8 or 52 weeks post-operatively. All patients went through the same post-operative re-hab protocol for knee replacement surgery with the hospital physiotherapists. Inclusion and exclusion criteria were as follows:

- *Inclusion criteria* – patients between the ages of 40 and 80; either awaiting knee arthroplasty or within one year of total knee arthroplasty; ability to walk 20 metres unaided; ability to sign informed consent.
- *Exclusion criteria* – walking with a frame or stick; post-operative complications such as active infection or DVT; neuromuscular conditions that could alter gait.

**Figure 1:**
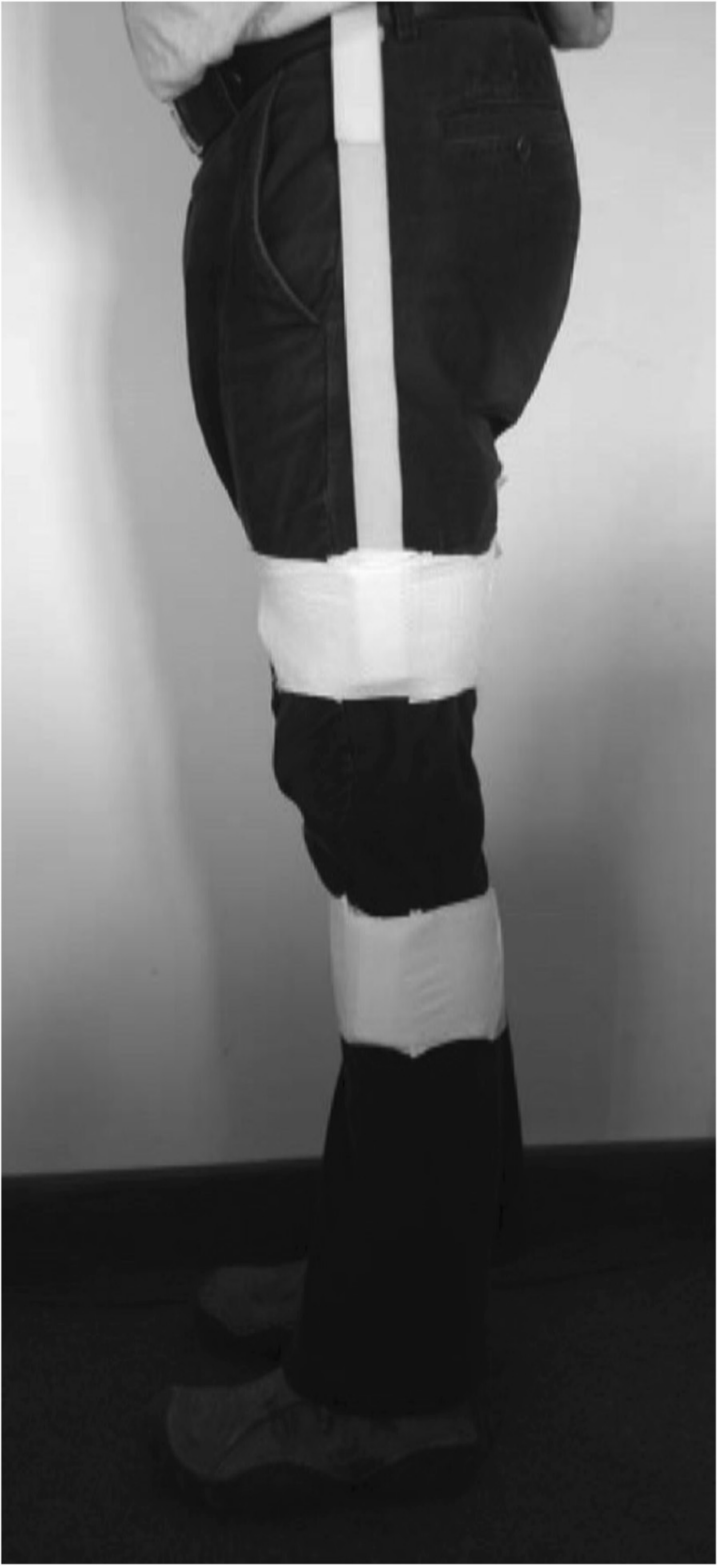
**Positioning of the IMUs using Velcro straps on each thigh and shank.** The straps could be applied outside clothing.

Motion sensors (GaitSmart, ETB, UK) were attached by Velcro straps, one to each thigh and shank (as shown in Figure 1); the sensors comprise three tri-axial accelerometers and three tri-axial gyroscopes. The thigh sensors were attached along the saggital plane of the thigh over the lateral aspect approximately 10 cm above the lateral joint line. The shank sensors were likewise attached over the widest part of the calf muscle taking different patient heights into consideration. Patients were then asked to walk for 10 metres along a corridor, turn round, and then walk 10 metres back. The sensors were then removed, and data downloaded to computer for calculation of thigh and shank sagittal and coronal angles, knee sagittal angles, and temporal descriptors of gait. A number of discrete parameters were then extracted from the data for a typical stride, and after preliminary inspection of the data, the following were selected for detailed analysis: knee range of motion during swing (knee_swing) and stance (knee_stance) phases; overall thigh sagittal ROM (thigh_sag); overall shank sagittal ROM (shank_sag); coronal thigh ROM (thigh_cor); coronal shank ROM (shank_cor); the difference in timing between the two peaks of thigh sagittal angle (t_p_diff); and stride duration (ave_dur). Oxford Knee Score questionnaires were completed pre-operatively and 52 weeks post-operatively, and passive range of motion was also recorded. Other data collected included: age and gender; medical history including previous surgery on the limbs; the type of prosthesis used in post-operative patients; surgical complications. In addition, 29 age- and gender-matched controls were measured for comparison with the knee arthroplasty patients. Ethical approval for the study was obtained from NRES Committee London City Road and Hampstead (Ref: 12/LO/0038). All participants gave written informed consent.

Differences between controls and the three patient groups were analysed with multi-variate analysis of variance (MANOVA) using SPSS Version 21; in cases of significant differences, the Bonferroni post-hoc test was performed. Further discriminant analysis of the data was performed to identify the most important variables in discriminating between the groups.

## Results

74 patients were recruited in total: 29 pre-operatively, 17 at 8 weeks post-operation and 28 at 52 weeks after operation. The mean age of the patients was 66.9 (10.7) years [mean (s.d.)]; the male:female ratio was 32:42. All surgery was performed with a medial peri-patellar approach using cruciate-retaining implants (Genesis II (n = 25); Triathlon (n = 7); PFC (n = 13)). There were 29 age-matched controls (M:F ratio 12:17), with a mean age of 68.1 (7.1). Pre-operative Oxford Knee Scores were 20.3 (7.7), 21.5 (8.6) and 20.1 (7.7) for the pre-op, 8 week and 52 week groups respectively; there were no significant differences between the three groups. The BMI for the controls was significantly lower that for the patients (26.1 (3.8) and 29.9 (4.7) respectively). There was no significant difference in the BMI for the three patient groups.

### Gait data

**Figure 2:**
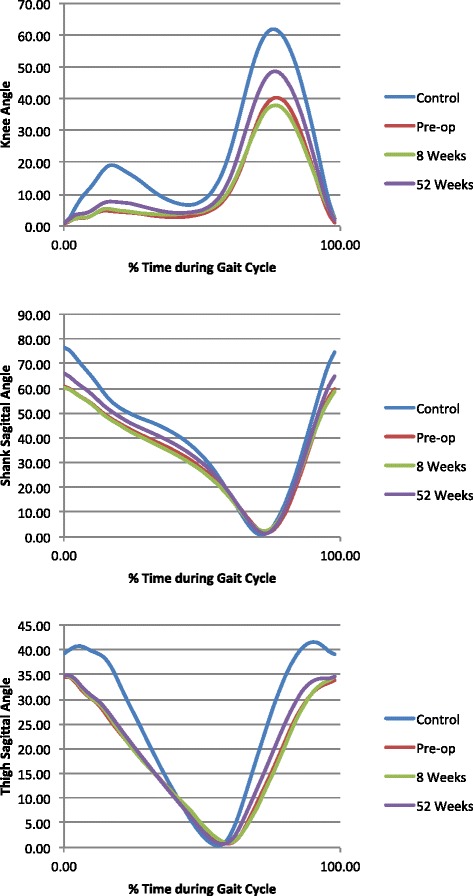
**Typical angles during gait cycle for knee, shank and thigh for the four groups in the study.**

Mean values of knee, shank and thigh angles during the gait cycle are shown in Figure 2. Inspection shows obvious differences between the curves. Mean values for the quantitative parameters extracted from the gait profile for both the operated and non-operated legs are shown in Table 1. MANOVA showed there was a significant difference in gait variables when comparing the patient groups and healthy age-matched controls (using Roy’s Largest Root: θ = 2.95, F(8,94) = 34.66, p < 0.001). Separate univariate ANOVAs showed significant differences between controls and the three patient groups for all variables except thigh coronal ROM.

**Table 1:** **Gait variables in the operated and non-operated legs for pre-op (n = 28) and 8-week (n = 17) and 52-week (n = 28) post-op, and age-matched healthy controls (n = 29); values are mean (s.d)**

		**Knee stance**	**Knee swing**	**Thigh sag ROM**	**Shank sag ROM**	**Thigh cor ROM**	**Shank cor ROM**	**T-P Diff**	**Stride Dur. (s)**
Op limb	Pre-op	**6.02** (3.41)	**42.51** (10.18)	**35.37** (6.39)	**61.65** (8.98)	**11.85** (6.43)	**12.51** (4.35)	**6.28** (7.28)	**1.31** (0.16)
	8 weeks	**6.23** (3.95)	**40.94** (12.83)	**36.78** (3.83)	**62.57** (10.25)	**13.03** (5.38)	**13.38** (4.73)	**8.59** (9.08)	**1.33** (0.23)
	52 weeks	**8.35** (3.72)	**50.64** ^**§**^ (7.78)	**36.40** (6.38)	**66.39** (8.34)	**11.94** (4.52)	**13.03** (6.09)	**10.79** (9.70)	**1.24** (0.18)
Non-op limb	Pre-op	**9.64** (5.97)	**47.46** (9.42)	**34.73** (6.79)	**62.85** (8.85)	**11.01** (4.97)	**14.42** (8.05)	**7.17** (5.25)	**1.31** (0.16)
	8 weeks	**10.15** (4.83)	**49.16** (7.68)	**34.67** (4.70)	**64.32** (8.16)	**11.57** (4.43)	**13.93** (6.94)	**9.06** (5.48)	**1.33** (0.23)
	52 weeks	**10.58** (4.43)	**50.74** (9.17)	**35.32** (5.79)	**65.35** (7.81)	**12.47** (5.77)	**13.89** (7.40)	**12.71** (7.59)	**1.24** (0.18)
Control		19.82* (4.92)	62.63* (5.77)	42.53* (5.77)	76.60* (5.80)	**11.89** (3.31)	17.29* (7.01)	16.55* (4.26)	1.07* (0.09)

(*indicates statistically significant differences between controls and patients; ^§^indicates a statistically significant change from pre-operative value; mean values are highlighted in bold typeD).

When comparing patient groups, knee_swing increased by almost 10° on the operated side at 52 weeks (p = 0.02); at this time there was very good symmetry during swing phase for the two limbs. Knee_stance was lower on the operated side at all three time points compared with the non-operated knee. Although there was a slight increase in knee_stance from pre-op to 52 weeks post-op, this was not statistically significant. Stride duration decreased slightly by 52 weeks, but this was not statistically significant (p = 0.053). There were no significant changes in any of the parameters for the non-operated leg.

As MANOVA showed that most gait variables were different when comparing controls and the patient groups, MANOVA was followed up with discriminant analysis to identify the most important factors discriminating between patients and controls. This analysis revealed three discriminant functions; the first discriminant function explained 95.5% of the variance, whereas the second explained only 3.7% of the variance and the third only 0.8%. In combination, these three functions significantly differentiated between healthy age-matched controls and TKR patients (ʎ = 0.222, χ^2^(24) = 144.50, p < 0.001), but removing the first function revealed that the second and third functions did not significantly differentiate between controls and patients.

**Figure 3:**
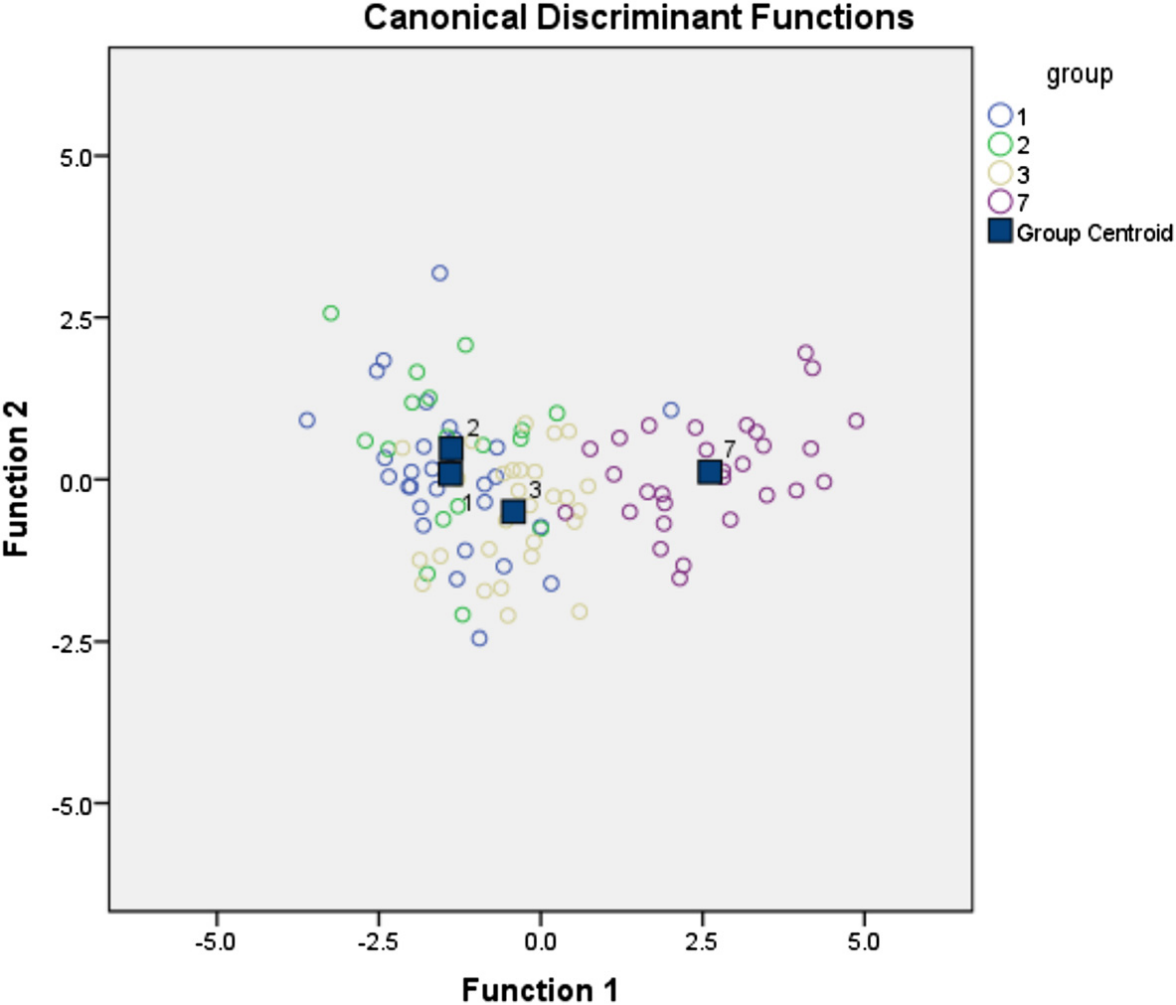
**Plot of the first two canonical discriminant functions.** Discriminant function 1 discriminates between TKA patients (groups 1 to 3) and healthy age matched controls (group 7). Discriminant function 2 discriminates between 8 week post-op patients (group 2) and 52 week post-op patients (group 3).

The discriminant functions are described in detail in Appendix A. A plot of discriminant functions 1 and 2 for all groups is shown in Figure 3. This shows that the first function discriminates the patient groups (1 to 3) from the control group (7). The controls have higher positive values on discriminant function 1, and knee_stance notably has the highest load (0.791) on discriminant function 1, indicating that this is the most important differentiator between the patients and controls. Thigh range has only a small load on this function (0.033), indicating that it is the least important discriminator between the controls and patient groups. Dimension 2 discriminates patient group 2 and 3. Patients group 2 have higher positive values on discriminant function 2. Knee_swing has the highest loading on discriminant function 2 (−1.018), indicating that it is the most important discriminator between patient group 2 and 3; the next most important factor is knee_stance with a loading of 0.526. For both discriminant factors, ave_dur had relatively low loading.

**Figure 4:**
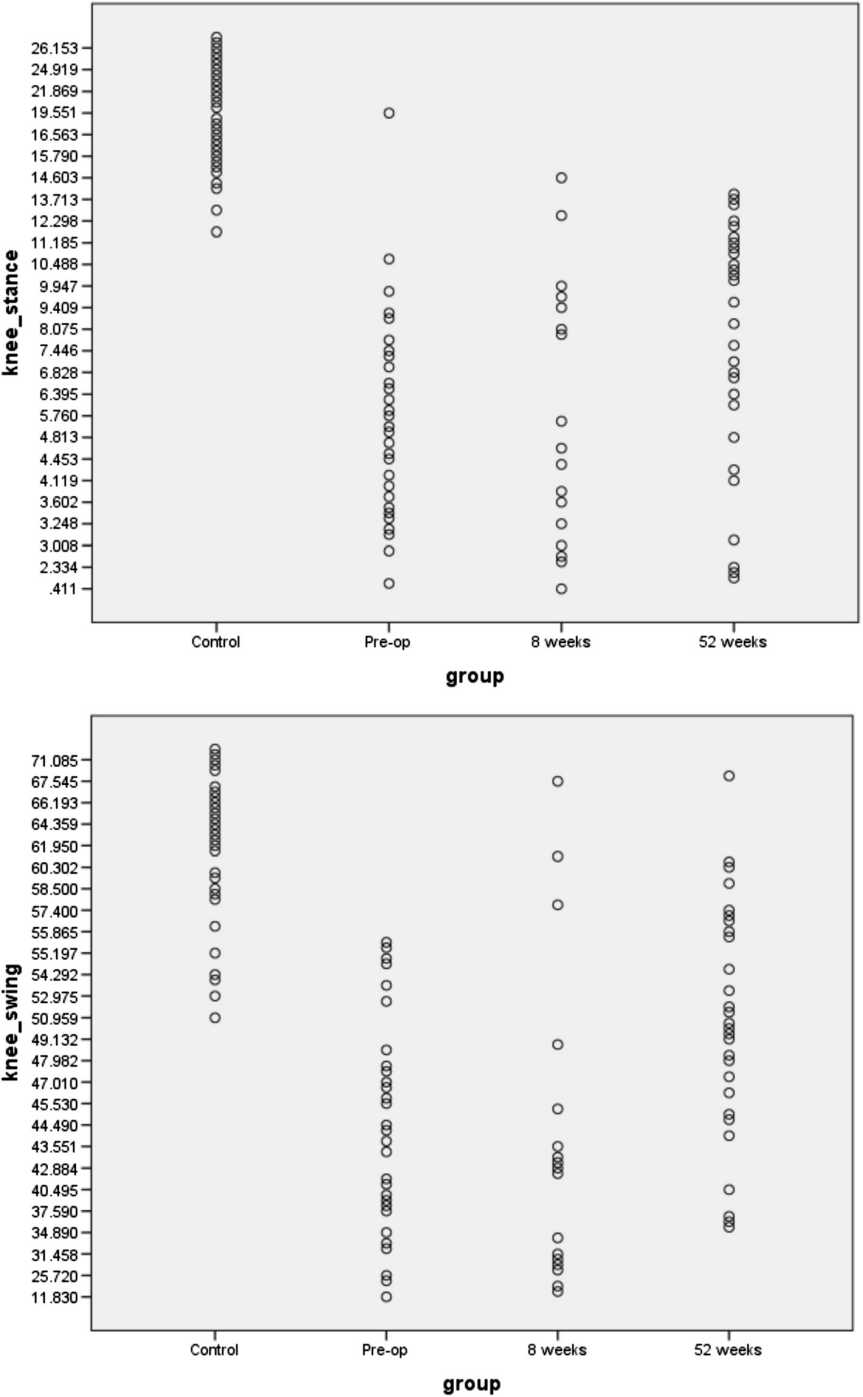
**Box plots for knee range of motion during stance and swing for controls, pre-op patients and post-op patients.** MANOVA showed that these two variables were the most significant in assessing the effects of surgery, and comparing patients with control.

As discriminant analysis has shown that knee_stance and knee_swing are the most significant variables when comparing all the groups, dot plots for knee_ swing and knee_stance are shown in Figure 4, for the controls, pre-op patients, and patients 52 weeks post-operatively. It can be seen that there is more overlap between knee_stance values in controls and post-op patients than for controls and pre-op patients. Only 1/29 pre-op patients were within the normal range, whereas 9/28 patients were within the normal range at 52 weeks (χ^2^ = 5.83).

### Clinical assessment

Pre-operative Oxford Knee Score was 21.2 (7.6) [mean (sd)], and for the 52-week post-operative group it was 38.1 (7.9). Passive knee range-of-motion was 103.8° (16.0°), decreasing to 99.7° (12.1°) at 8 weeks post-operatively, and then increasing to 107.4° (10.7°) at 52 weeks. There was only a weak correlation between the passive range of motion of the knee and knee range measured dynamically during gait (r^2^ = 0.147) and knee stance flexion (r^2^ = 0.03).

## Discussion

We have performed a cross-sectional study of a population that is representative of people in our hospital undergoing knee replacement surgery. We have assessed gait with regards to knee range of motion prior to and after knee arthroplasty surgery in a bid to understand if this improves with surgery. In so doing we have investigated the feasibility and utility of performing gait assessments in a busy clinical setting, to better understand the potential of such measurements as a clinical outcome measure.

In assessing the effects of surgery, a statistically significant increase of knee sagittal range of motion during swing was observed. In comparison with the healthy active age-matched control group, all sagittal plane variables were significantly different in the TKA patients, in addition to stride duration, which was 25% slower than controls. However, subsequent discriminant analysis identified that sagittal plane knee ROM in stance and swing were the two most important variables in discriminating between the controls and TKA patients, despite the implied difference in walking speed between the groups; previous studies have indicated that knee movement during stance is probably independent of gait speed [23]. We have previously shown that IMUs can be used to discriminate between controls and subjects with early OA from the measurement of knee ROM in stance [21], which is in agreement with other studies [24,25]. Thus TKA patients maintain the characteristics of OA gait after their surgery, even though pain has reduced. Their passive range of motion has increased slightly, so patients were using only a fraction of their potential knee movement during gait [17].

The main effect of surgery was to result in a higher knee range of motion during swing. Although motion of the swing leg is likened to that of a compound pendulum, simulations have shown that reduced knee flexion angle during swing may be caused by overactivity of the rectus femoris, weakened hip flexors or a large knee flexion velocity at toe off [26]. Subsequent analysis by the same group showed that knee flexion velocity at toe-off contributed most to peak knee angle (30°) [27]. In our study, peak knee angular velocity was 191 degrees/s pre-operatively and increased to 231 degrees/s at 52 weeks post-operatively. We suggest that this difference in angular velocity could contribute to the differences in peak knee angle seen after surgery; it is possible that pain relief provided by surgery at 12 months contributes to the faster knee angular velocity.

Peak knee flexion in stance corresponds with the time of peak knee flexion moment, and this moment has been shown to be reduced in TKA patients [11]. In their musculoskeletal modelling, the authors showed that the quadriceps contribute significantly less to the extension moment developed about the knee during early stance in patients with TKA, which they described as a “quadriceps avoidance” gait pattern [11]. Patients may develop such a gait pattern during the progression of OA in order to reduce load, and therefore pain, on the knee, but this gait pattern appears to be maintained post-operatively.

Changes in muscle activation patterns have been reported in TKA [28,29], and could explain the limited knee angle in stance post-operatively. Reduced quadriceps force and volitional activation have also been reported [30]. Prolonged muscular co-contractions of rectus femoris, hamstrings and tibialis anterior during stance have been observed, and co-activation of these muscles may stabilise the knee during stance phase [28]. Our patients showed relatively little change in knee angle during stance after the initial maximum, suggestive of co-contraction of antagonistic muscles, perhaps required for knee stabilisation.

It is, however, interesting to consider what should be expected as an outcome after knee replacement. Although knee movement improves, the mean values for the knee ranges of motion in both swing and stance phases at 12 months are still >2 SDs below the means for an active healthy control group; mean stride duration is also significantly below normal. Although mean values for gait parameters were significantly reduced in all patient groups compared to controls, it is interesting to inspect individual values; only 1/29 patients were within the normal range for knee stance flexion pre-operatively, whereas 9/28 were within the normal range 12 months post-operatively (as shown in Figure 4). These data indicate that good functional outcome is possible. It would therefore be very interesting to investigate the reasons why about one third of TKA patients can achieve good functional outcome, but two thirds have poor outcome. It would also be important to establish whether directed rehabilitation can improve outcome, and at what stage this can most effectively be implemented.

Other studies have investigated the use of gait as an outcome measure in the assessment of knee replacement [31,32]. The authors presented a visual classification system, classifying patients as “dominant normal”, “non-dominant normal”, “non-dominant OA”, and “dominant OA”, based on the position of a summary score in a simplex plot [32]. Pre-operatively 8/9 patients were classified as “dominant OA” on the basis of gait measurements, and 12 months post-operatively 7/9 patients were classified as “dominant OA”, indicating gait characteristics similar to patients with severe OA [32]. This classification system used a full opto-electronic gait system and a complex algorithm to classify patients, which could not be used as a matter of routine in a busy out-patient department. The simple analysis of knee flexion in stance described in this paper is feasible for routine clinical use, and appears to be just as effective in evaluating outcome.

Although we have used a measurement technique that can be used in an out-patient clinic, we have still evaluated patients in a simple environment that requires walking in a straight line on a level surface. In order for patients with knee OA, both before and after surgery, to maintain a mobile healthy lifestyle, they need to negotiate more complex terrain, involving gradients, steps, and uneven surfaces, and require to share that space with other users [18]. The degree to which individuals utilise the opportunity for activity will depend on their capabilities. The capabilities model stresses that the overall objective for a person is to be able to undertake the activities they wish to do, and their ability to achieve this depends on the capabilities required by the activity and its associated environments and the capabilities provided by the individual [33]. We have demonstrated that the provided capabilities of TKA patients are impaired, so in order to improve mobility of these patients the gap between provided and required capabilities needs to narrow. Objective measurements of function within a complex environment can inform developments to improve mobility, for rehabilitation professionals, implant designers, and those involved with the design of the built environment and transport infrastructure.

## Conclusions

We have taken measurements of gait pattern in groups of patients, before and after TKA, in out-patient clinics using IMUs. The main factor differentiating all patients from age-matched controls was knee flexion during stance, a general characteristic of osteoarthritic gait that is maintained after surgery. Twelve months after TKA, knee flexion in swing was higher than in pre-operative patients and gait patterns were symmetrical. Stride duration was not different after surgery, being around 25% longer than that of controls for all three TKA groups. The potential exists to identify patients who may benefit from additional rehabilitation, and monitor their progression post-operatively.

## Appendix A

*Discriminant function 1 = 0.791*knee_stance + 0.393*t_p_diff + 0.365*knee_swing −0.246*thigh_cor −0.232*shank_sag −0.193*ave_dur +0.109*shank_cor + 0.033*thigh_sag*

*Discriminant function 2 = −1.018*knee_swing +0.526*knee_stance + 0.372*thigh_sag +0.202*shank_sag + 0.190*thigh_cor +0.183*shank_cor +0.170*ave_dur −0.168*t_p_diff*

*Discriminant function 3 = 0.814*shank_sag + 0.755*t_p_diff - 0.466*knee_swing −0.411*knee_stance + 0.253*thigh_cor +0.078*shank_cor −0.063*ave_dur −0.027*thigh_sag*
